# Domestic water consumptions and associated factors in rural household of Harari region, Eastern Ethiopia; A cross sectional study

**DOI:** 10.3389/fpubh.2024.1395946

**Published:** 2025-01-16

**Authors:** Bushra Yusuf Ahmed, Sina Temesgen Tolera, Liku Muche Temesgen, Abraham Geremew

**Affiliations:** ^1^Job Creation, Harar, Ethiopia; ^2^Department of Environmental Health, College of Health and Medical Sciences, Haramaya University, Harar, Ethiopia

**Keywords:** domestic, per capita, water consumption, rural, Ethiopia

## Abstract

**Background:**

Sustainable rural water supply management requires accurate estimations of water consumption and understanding factors influencing consumption. Climate change exacerbates challenges, especially in developing countries with high poverty and limited access to clean water. Ethiopia has the lowest municipal water supply in Africa. The residents of the Harar region currently get water once a week or two weeks for just a few hours, which is very inadequate for everyday household activities like drinking, cooking, cleaning utensils, washing clothes, etc. Despite global efforts to identify the factors that affect domestic water consumption, those related to domestic water use in rural regions have not been sufficiently studied, particularly in rural woredas of Harari region, Ethiopia.

**Objective:**

To assess the domestic water consumption and associated factors at household level in rural woredas of Harari region, Eastern Ethiopia from October 29, 2022 to November 21, 2022.

**Method:**

A community-based cross-sectional study was conducted on 408 households using stratified sampling techniques, collecting data on socio-demographic and water use characteristics. Epi-Data and SPSS were used for data analysis. Descriptive analysis was used to determine average total and per capita water consumption, while a multiple linear regression model allowed for the identification of associated factors and the prediction of water consumption with statistical significance.

**Result:**

Daily water consumption was found to be 103.3 L/hh/d and 17.6 L/c/d. The associated factors included the main source of water, time of collection, household size, wealth status, water price, and frequency of gathering water.

**Conclusion:**

Thus, the provision of an adequate quantity of water for rural households should be given attention for maintaining human wellbeing, and the consideration of socio-economic factors affecting per capita water consumption is desirable in the planning and implementation of proper water demand management strategies.

## 1 Introduction

Water is a vital life-sustaining component, and inadequate availability can lead to disease spread (1). Domestic water consumption includes drinking, food preparation, washing, cleaning, bathing, and other domestic tasks. The World Health Organization defines domestic water as water used for all domestic purposes, including consumption, bathing, and food preparation (2).

Domestic water supplies significantly impact hygiene and public health. A minimum of 25 liters per day is considered a basic level of service. International organizations and water providers should adopt a Basic Water Requirement (BWR) of 50 liters per capita per day to meet four domestic basic needs: drinking, sanitation, bathing, and cooking. This requirement should be guaranteed to all humans, making clean water a fundamental human right (3).

Countries worldwide face challenges in maintaining reliable water supply, exacerbated by global climate change. Growing populations and limited cost-effective options make it difficult to augment water supply. Different stages of development lead to varying water supply and consumption statuses, with developed countries using more water than less developed ones. This issue is expected to persist in the future (4, 5). In most countries, there are populations whose water is derived from household sources, such as private wells and rainwater. In households using non-piped water supplies, appropriate efforts are needed to ensure safe collection, storage and perhaps treatment of their drinking-water (6).

In developing countries with high poverty and limited access to clean water, policymakers must design urban water systems that cater to both connected and non-connected households (7). Accurate daily water consumption is crucial for community planning and designing water supply and waste treatment facilities. Inaccuracies can lead to inadequate service (8, 9). The water supply must meet accessibility, quality, quantity, and effective wastewater disposal demands, all at a reasonable cost (10).

Despite having a large amount of fresh water resources, Ethiopia has the lowest municipal water supply in Africa. With twelve major lakes and twelve river basins, Ethiopia has an annual renewable fresh water resource of 122 billion cubic meters per year and a groundwater potential of 2.69 BCM. However, population growth is causing a decline in the per capita water resource potential for 2010 is estimated at 1,500 cubic meters per year, close to the World Bank’s water scarcity threshold of 1,400 m^3^/year per capita than of the estimated for 2010 of 1,500 m^3^/year per capita (9, 11, 12).

Further, a study in Shambu, western Ethiopia, found that the total water demand was 567,648 m^3^/year, or 18 L/s, significantly higher than the total water supply of 252,288 m^3^/year, or 8 L/s (13). Similarly, a finding from Yejebu town, northern Ethiopia, addressed that average daily water consumption in 2018 was 16 L/c/d and is expected to reach 36.7 L/c/d by 2040 and, indicating unbalanced water production and demand (9).

As populations grew, the challenge to meet user demands also increased. In Ethiopia, both governmental and non-governmental development agents have been involved in order to enhance the coverage of potable water supply in different parts of the country. But, the coverage of the service in the country still lags (14). According to Ethiopia Demographic and Health survey report, only 57% of rural households have access to an improved source of drinking water, as compared with 97% of urban household’s (15).

Moreover, WHO recommends 50–100 L of water per capita per day (LCPD) to meet domestic needs such as personal hygiene, washing and cleaning. But in Ethiopia context the amount is very low. In Nakemte town, East Wollaga zone of Ethiopia, daily per capita water is 15.26 liters for different domestic activities (16), less than 20 L/c/d in three urban areas of Addis Ababa, namely Taklehaimanot, Merketo and Biherestige (17).

Previous research on domestic water consumption in rural communities has been influenced by factors such as water source, walking distance, and waiting time (1, 18–20). These studies have primarily focused on urban residents, highlighting the need for further investigation into the determinants of water consumption in rural areas to contribute to existing empirical evidence and assess associated factors in rural woredas of the Harari region.

The purpose of this study is to investigate the determinants of domestic water consumption in rural communities of the Harari region, Ethiopia, and to identify the factors that influence water use patterns in these areas.

## 2 Materials and methods

### 2.1 Study area, period and design

The study was conducted in rural districts of Harari regional state, specifically in Sofi, Dire Teyera, and Erer Woreda, from October 29, 2022, to November 21, 2022, in Ethiopia, by using a community-based cross-sectional study design. Sofi is located 510 km east of Addis Ababa and has a population of 49,046, with 25,630 males and 23,416 females. It has 7 kebeles and 11 health centers. Dire Teyera is 7 km north of Harar town and 517 km from Addis Ababa. It has six rural kebeles and a total household population of 10,565. Erer Woreda is 10 km north-east of Harar city and 519 km from Addis Ababa. It contains four kebeles and 6,019 households, with 24,318 total populations.

### 2.2 Source and study population

The study involved all rural households in Harari regional state, selected systematically from eight randomly selected kebeles.

### 2.3 Inclusion and exclusion criteria

The study involved over 18 years old residents of Sofi, dire teyara, and Erer Woredas, responsible for water management, excluding heads involuntary information providers.

### 2.4 Sample size determination

#### 2.4.1 Sample size determination for first objective

The study used a single population formula to determine the required representative sample size based on the following assumptions: margin of error 3, confidence level of 95%, and contingency for non-response rate of 10%. The average domestic water consumption of households in Dire Dawa is 64.6 L/c/d, with a standard deviation of 26.99 (21). The sample size of 342 was obtained for the first objective.

Formula.


$$n={\left(Z\alpha /2\right)}^{2}\;{\left(\sigma \right)}^{2}/{\left(d\right)}^{2}$$

Where:

- n is the required sample size.
- Z { α /2} is the standard normal variable at *α*/2 (e.g., 1.96 for a 95% confidence level).
- *σ* is the population standard deviation.
- d is the margin of error.

#### 2.4.2 Sample size determination for second objective

Epi-Info was used to calculate the sample size, with a power of 80% and a 95% confidence level of 1.96(z). A study in Dire Dawa town found that households using non-pipe-connected water sources used inadequate amounts of water (57.4%) compared to pipe-connected sources (42.6%), with a statistically significant difference (*p*-value <0.05) (21). The sample size of 385 was obtained for the second objective.

Formula.


$$n=\left[\left(p_{1}q_{1}+p_{2}q_{2}\right)\;f\left(\alpha ,ß\right)\right]/{\left(p_{1}-p_{2}\right)}^{2}\text{.}$$

Where:

- n is the required sample size.
- *α* (\alpha/2) is the standard normal variable at α/2 (e.g., 1.96 for a 95% confidence level).
- *β* (beta) is the standard normal variable at β (e.g., 0.84 for 80% power).
- p1 is the proportion in Group 1.
- p2 is the proportion in group 2.
- q1 = 1 - p1
- q2 = 1 - p2.

Finally, the sample size estimation for the second objective was 385, which was used because it provided a larger sample size when compared to the first.

### 2.5 Sampling procedure

Stratified sampling technique was used. Harari regional state has three rural districts, Sofi, Erer and Dire Teyera Districts/woredas. Firsly, eight kebeles were selected randomly from three districts. Finally, the required household samples were proportionally allocated to the selected eight kebeles by using systematic sampling (Supplementary Figure 2).

### 2.6 Data collection methods and procedure

Data were collected using a checklist and a pretested, closed-ended, structured questionnaire. Data was collected by two trained B.Sc. Environmental Health professionals and supervised by the principal investigator and water office expert. Data was collected through the administration of a pre-tested, structured questionnaire of interviews among the selected households. The questionnaire was prepared in English from a review of literature (1,12, 22 and 28) and then translated into the Afan Oromo language. The dependent variable, per capita daily water consumption, was obtained by asking the total daily water consumption of the household for different uses and dividing it by the number of people currently living in the family.

### 2.7 Study variables

#### 2.7.1 Dependent variables

Per capita water consumption.

#### 2.7.2 Independent variables

The independent variables in these studies are: - Age, family size, wealth, education status, gender, occupation, Access to appliances such as showers, toilets, Primary Source of water, price of water time of fetching and distance from sources.

### 2.8 Operational definition

**A kebele** is the smallest administrative unit in Ethiopia; it is comparable to a ward, neighborhood, or small town.

**Per capita water consumption** is the amount of water used by person per day (22).

**Water Demand** is the amount of water that would be consumed if the water was free of charge and available in unlimited quantities (22).

**The Wealth Index** is constructed by combining various asset indicators, such as ownership of assets like cell phones, televisions, and refrigerators, into a single score.

**Water consumption** is the actual amount of water consumed (22).

### 2.9 Data quality control

First, the questionnaire was developed from previous research and tested for reliability and validity. It was translated into Afan Oromo and given intensive training to data collectors. A pretest was conducted on 10% of the total sample size one week before data collection on Sofi Kebele. Data was collected through questionnaires with the household wife or fetcher. Amendments were made based on the pre-test results. Questionnaires were checked for completeness by both data collectors and supervisors before returning from the field and before data entry. The questionnaire was adapted for consistency and consistency.

### 2.10 Methods of data analysis

The data was manually checked, cleaned, and entered using Epi Data. The data was then exported to SPSS for analysis. Descriptive statistics, including mean, standard deviation, and percentages, were utilized to analyze the characteristics of dependent and independent variables. A multiple regression model was employed to assess the determinant factors of per capita water consumption. Normality and uniformity were tested, and multicollinearity was checked using the variation inflation factor (VIF) and tolerance value. The model’s tolerance range is 0.403–0.992, with a minimum value of 0.20 is recommended (23).

## 3 Results

### 3.1 Socioeconomic characteristics of respondents

A total of 383 households responded to this study, making 99.5% response rate. The result revealed that majority (95.6%) of respondents were female as by virtue of their traditional role in provision of water for households and data had been collected during crop harvesting season when farmers were busy on field works, so that females stayed to be interviewed. The mean age of the respondents was 31.73 years with standard deviation of 5.29 years with the range of 21 up to 60 years. Concerning religion, 98.7% of the household head were Muslim while the rest 1.3% were Christian Orthodox.

More than 90% of the respondents have no education while, 7.3% followed Primary education or above educational levels. The average family size of the households is 6 members and ranges of 1 to 14 members within a single household. The other socioeconomic survey result illustrated in (Table 1) was occupation of study participant, 98.2% are Housewife, 0.5% is student, 0.6% is daily laborer and merchant, 0.8% is government employed. The principal component analysis was used to assess the economic status (wealth Index) of households based on the assets they hold and the descriptive statistics result indicates that, about 18.8, 21.9, 18.5, 21.7, 19.1% of Households are in Lowest, Middle lowest, Middle, Middle Highest and Highest wealth quantile, respectively.

**Table 1:** Socio demographic and economic characterists of sampled househods in rural Harari region, Eastern Ethiopia, November 2022.

Variables	Catagories	Frequency	Percent
Sex	Male	17	4.4
	Female	366	95.6
	Total	383	100.0
Religion	Muslim	378	98.7
	Orthodox	5	1.3
	Total	383	100.0
Educational level	No education	355	92.7
	Primary	19	5.0
	Secondary	7	1.8
	Higher education	2	0.5
	Total	383	100.0
Occupation	Housewife	376	98.2
	Student	2	0.5
	Daily laborer	1	0.3
	Merchant	1	0.3
	Other	3	0.8
	Total	383	100.0
Wealth index	Lowest	72	18.8
	Middle Lowest	84	21.9
	Middle	71	18.5
	Middle Highest	83	21.7
	Highest	73	19.1
	Total	383	100.0

### 3.2 Housing ownership, sanitation and hygiene facilities

Among the households surveyed, 314 (82%) had toilet facilities, and 249 (65%) used water for toilets. Regarding shower rooms, respondents asked whether they had a shower room with a tap or not, and only 15 (3.9%) had owned one (Table 2).

**Table 2:** Shower room and toilet in rural Harari region, Eastern Ethiopia, 2022.

Variables	Categories	Frequencies	Percent
Shower room with tap	Yes	15	3.9
	No	368	96.1
	Total	383	100.0
Toilet availability	Yes	314	82.0
	No	69	18.0
	Total	383	100.0

### 3.3 Domestic water consumption and its characteristics

#### 3.3.1 Sources, time of fetching, distance from source and price of water

**Figure 1:**
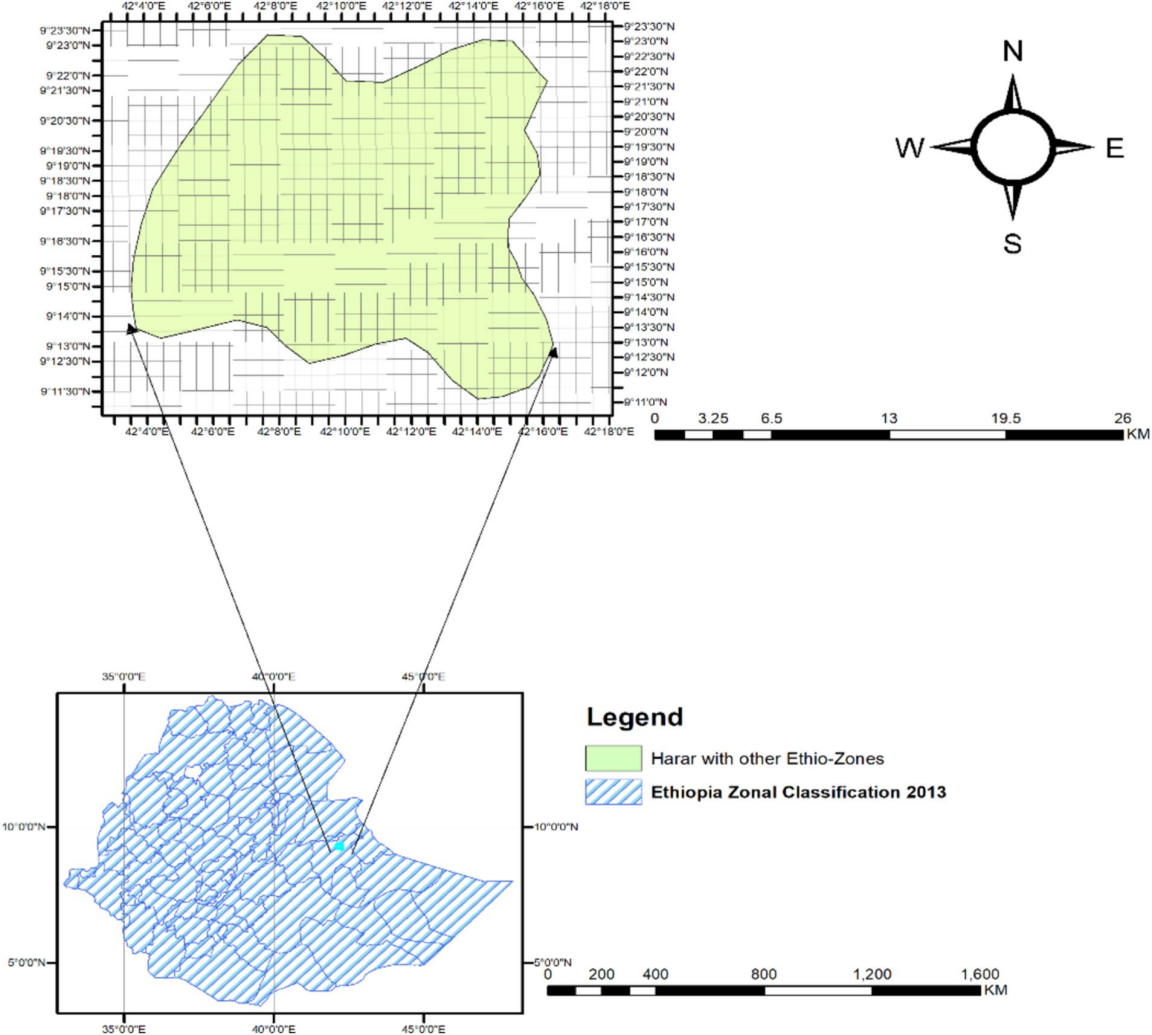
Location map of study areas (sub districts) in Harari region in the map of Ethiopia.

**Figure 2:**
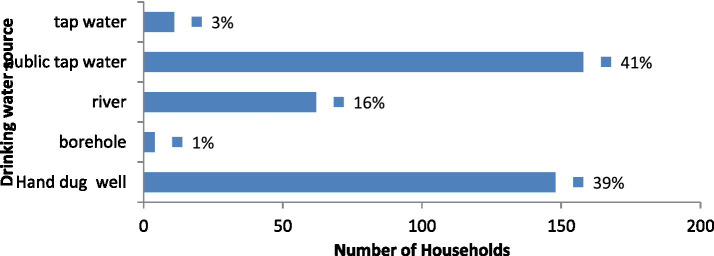
Main source of domestic water supply in rural Harari region, Eastern Ethiopia, November 2022.

The study found that a majority (41.3%) of households in the study area rely on public tap water for domestic use (Figure 1), with hand-dug wells being the second largest source (38.6%), followed by river water (16.2%), and the remaining 3.9% use other sources (tap water and bore holes). In addition, out of 383 families surveyed, 69 (18%) had one or more alternative sources of water, while 82% did not, according to a study on households’ water supplementation strategies (Figure 2).

Out of the total sampled households, 212 (55.4%) reported continuous water supply, while 25.3, 0.8, 11, and 7.6% received water more than once a day, once a week, once every two weeks, and more, respectively. About half of households (47.5%) had access within 30 min or less. 33.9% of households paid for water, while 66.1 percent did not. The average monthly price for those who paid was 1,057 birr (Table 3).

**Table 3:** Frequency of water supply and time of collection for househods in rural Harari region, Eastern Ethiopia, November 2022.

Variable	Categories	Frequency	Percent
Water supply	24-h supply	212	55.4
	More than once a day	97	25.3
	Once a day	3	0.8
	Once in week	42	11.0
	Once in two weeks and more	29	7.6
	Total	383	100.0
Duration of time to fetch water and return home	Less than 15 min	80	20.9
	Less than 30 min	102	26.6
	More than 30 min	56	14.6
	More than 1 h	145	37.9
	Total	383	100.0

The survey revealed that 43.3% of respondents regularly received water from main sources, while 56.7% identified interruptions or shortages in the area, forcing them to purchase water from commercial sources or find alternative sources. The shortage becomes acute during summer, with most people using water from rivers (8.4%), hand-dug wells (2.3%), venders (18.3%), and fetching by hand (7.8%). Some respondents (20%) waited for water service to return (Table 4).

**Table 4:** Water source during shortage time of househod in rural Harari region, Eastern Ethiopia, November 2022.

Variables	Categories	Frequency	Percent
source of water during shortage availability	No shortage	166	43.3%
	Dug well	9	2.3%
	Erer river	32	8.4%
	Fetch by Hand	30	7.8%
	Nowhere	56	14.6%
	Wait for public tap	20	5.3%
	Vender	70	18.3%
	Total	383	100.0

#### 3.3.2 Daily domestic consumptions of water

**Figure 3:**
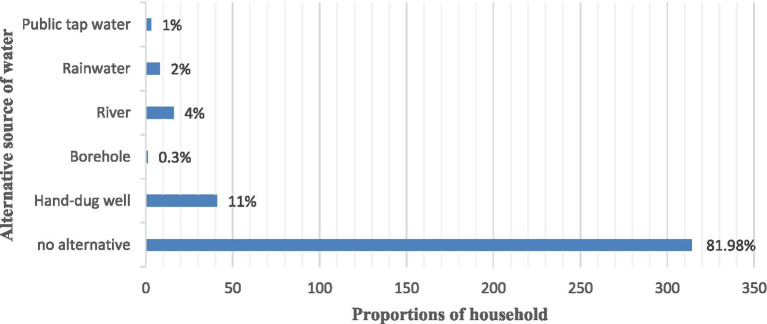
Alternative source of water in rural household of Harari region, Eastern Ethiopia, November 2022.

The study revealed that 383 households in the study area consumed 39,460 liters of water daily, with an average of 103.3 liters and a standard deviation of 40.3. The dependent variable, per capita daily water consumption, has an average of 17.6 liters per day and a standard deviation of 6.9. The majority of water was used for cooking and food preparation (29%), drinking (26%), bathing and laundry (23%), toilets (13%), livestock (8%), cleaning houses (0.76%), and gardening (0.15%). In addition, the missing values indicate that 30% of households used water for bathing and laundry at the source, 22.4% did not have livestock, 49% drank from livestock at the source, and 45% did not use water for toilets (Figure 3).

### 3.4 Factors affecting consumption of water

The study conducted a multiple linear regression analysis to determine the determinants of average per capita water consumption, revealing an *R*^2^ value of 0.591, indicating that 59.1% of the variance in this area was explained by the model’s explanatory variables. The study found a negative association between family size and per capita water consumption, with a coefficient of 1.446, indicating that an increase in household size leads to a decrease in per capita water consumption by 1.446 litters. Households with a frequency of water supply of more than once a day (2.701) and once every two weeks (5.127) had significantly lower levels of per capita water consumption than those with a 24-h continuous supply (*p* < 0.05).

Regarding the effect of time collection, households with a time of collection less than or equal to 15 min have a significantly 5.38 times higher level of per capita water consumption than those who had a time of collection of more than 1 h at the water source with a 95% level of confidence interval. Households with the main source public tab have a significantly 2.948 lower level of per capita water consumption than households that used hand-dug well water sources, with a 95% level of confidence interval (ß-coefficient = −2.948, *p* = 0.016).

Further, the wealth index of households with the 4th wealth index dummy has a significantly 1.436 litters lower level of per capita water consumption than households with the 5th wealth index, with a 95% confidence interval. The test result also shows that the average monthly water price was positively associated with the quantity of water consumed (*ß*-coefficient = 0.004, *p* = 0.000) (Table 5).

**Table 5:** Multiple regression result for factors associated with percapita water consumption in rural Harari region, Eastern Ethiopia, November 2022.

Coefficients^a^							
Variables	Unstandardized coefficients		Standardized coefficients	*t*	Sig.	Collinearity statistics	
	*B*	Std. Error	Beta			Tolerance	VIF
(Constant)	43.507	8.135	-	5.348	0.000	-	-
Age	−0.031	0.090	−0.025	−0.340	0.735	0.693	1.442
Price	0.004	0.001	0.431	4.987	0.000*	0.520	1.921
Shower room	−3.004	2.180	−0.098	−1.378	0.171	0.767	1.304
Toilet	−1.564	1.135	−0.106	−1.377	0.171	0.657	1.522
Gender	−5.046	2.556	−0.151	−1.974	0.051	0.664	1.506
River source	−4.768	4.937	−0.065	−0.966	0.336	0.863	1.159
Public tap source	−2.948	1.205	−0.218	−2.446	0.016*	0.488	2.049
Houseld tap source	−2.135	2.207	−0.089	−0.967	0.336	0.464	2.155
Primary education	4.339	4.873	0.059	0.890	0.375	0.885	1.130
Secondery education	0.094	2.057	0.004	0.046	0.963	0.589	1.699
Higher education	5.109	3.988	0.098	1.281	0.203	0.666	1.501
Merchant occupation	−2.273	4.900	−0.031	−0.464	0.644	0.876	1.142
More than once a day	−2.701	1.246	−0.201	−2.167	0.032*	0.452	2.213
Once a day	−1.745	3.244	−0.041	−0.538	0.592	0.676	1.479
Once in two weeks	−5.127	1.804	−0.269	−2.842	0.005*	0.434	2.305
Family size	−1.446	0.191	−0.549	−7.592	0.000*	0.746	1.341
Less than or equal to 15 min	5.380	2.074	0.260	2.594	0.011*	0.388	2.576
less than 30 min	1.206	1.794	0.050	0.672	0.503	0.702	1.424
More than 30 min	0.908	1.177	0.062	0.772	0.442	0.612	1.634
Poorest	−2.381	1.725	−0.158	−1.381	0.170	0.297	3.365
Middle poorest	−2.485	1.805	−0.153	−1.377	0.171	0.317	3.153
Middle	−2.371	1.641	−0.157	−1.445	0.151	0.328	3.047
Middle wealthest	−1.436	1.580	−0.092	−1.990	0.001*	0.380	1.628

*The *p*-values in the bold indicate that the constant (intercept)/variable is significant at the 5% significance level.

## 4 Discussion

The daily domestic water consumption by surveyed households in rural Harari Region has been estimated at 39,460 liters. The finding that the average consumption of water per household per day was 103.3 liters and the average consumption per capita per day was 17.6 liters is comparable with studies conducted in different parts of the country (11, 16, 21, 24). But much lower than the findings reported in India by (25), in China by (26) and in Nigeria by (27), which were 117 L/c/d, 56.2 L/c/d, and 92.7 L/c/d, respectively. Furthermore, it is clear from our findings that the average water usage per person is significantly beyond the intermediate access threshold of 50 L/c/day, the minimum quantity of water recommended by the World Health Organization (WHO) to ensure Adequacy for health needs (2).

The study found that six explanatory variables significantly influenced per capita water consumption, explaining 59.1% of the variance in this area, with an explanatory power of 0.591. This value is lower compared with study conducted in Ethiopia (28) (*R*^2^ = 0.92), but different from others findings in the country (21) and by (11) (*R*^2^ = 0.399), in China (26)(*R*^2^ = 0.37). The low explanatory power of the factors (*R*^2^ values) in these studies suggest that there are much more underlying factors impacting on water use that are not yet to be discovered. In addition, numerous studies have evaluated the factors that influence water consumption rates, either directly or indirectly. One such study examined the impact of price and non-pricing-related factors on the water demand of multiple selected households from ten different nations (29).

Furthermore, the study reveals that water consumption at the household level is highest for cooking and food preparation, accounting for 29.0% of total consumption. Other water uses include drinking (26%), bathing and laundry (23%), toilet (13%), livestock domestication (8%), house cleaning (0.76%), and gardening (0.15%). However, by average consumption bathing and laundry contain highest value of 33.8 L followed by cooking and food preparation (30.3 L) and cleaning house contains the least (20 L). This finding is similar with studies conducted in east wollaga zone of Ethiopia by Ali and Terfa (16) and in Nigeria by Sharma and Bereket (30).

**Figure 4:**
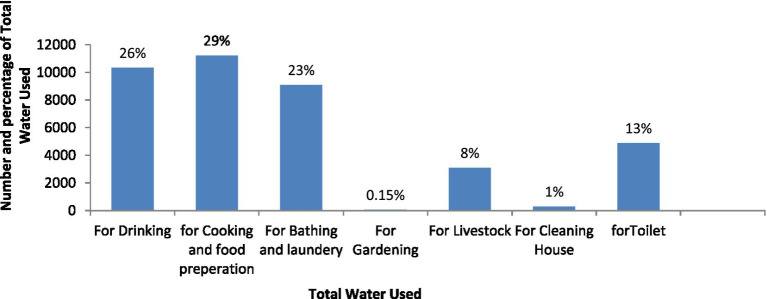
Number and percentage of total water used for different purposes by househod in rural Harari region, Eastern Ethiopia, November 2022.

In this study, public tap main source of water supply was significantly associated with per capita domestic water consumption with *ß*-coefficient = −2.948 and *p* = 0.016. It indicated that those households with main source public tab have significantly 2.948 litters lower percentage changes in their level of per capita water consumption than households who used hand dug well water source. The reason was unreliability of improved water sources. Tap water and public tap in study area were not available all the time. Sometimes the intermittent system waits for more than one weak and peoples obligate to use unimproved water sources (Figure 4).

Further, households with a collection time of less than or equal to 15 min at the water source had a significantly higher percentage change (5.38%) in their per capita water consumption than those with a collection time of more than 1 h. This is due to the study considering collection time as a dummy variable, using more time as a base category, and inferring a lower collection time. This study is consistent conducted in Nigeria and in Ethiopia (28, 31).

Furthermore, household size negatively impacts per capita water consumption, with an unstandardized coefficient of −1.495. For every unit increase in household size, per capita water consumption decreases by 1.49 litters in the study area. The study done Ethiopia suggests that larger households collect more water than individual households, leading to a positive relationship between household size and total water consumption. However, per capita water consumption decreases with larger household sizes due to access and adequacy issues, where all household members share less water than individual access per day (32). This result also inline with finding from Nigeria and Ethiopia (32, 33).

Additionally, the study found that the average monthly water price significantly impacts daily water consumption per capita and is positively correlated with water usage. However, the coefficient’s sign is positive (0.004), contradicting the expectation. The consumption analysis considers water consumption from all sources, with price increases due to alternative sources. This results in lower price elasticity than a source-specific analysis, as it captures expected inter-source consumption substitution in response to price changes. The study suggests that alternative resources are more prevalent in water consumption. This findig is in line withstudys done in Ethiopia (21). However, findings from Nigerian reports show that households that pay for water consume less compared to those with in-house access or free water (34).

The study reveals that the wealth index is significantly associated with daily per capita water consumption. Households with the 4th wealth index have significantly lower changes in per capita water consumption (1.436%) compared to those with the 5th wealth index, with a 95% level of confidence interval, indicating that the wealthiest households consume more water than the poorest. This is consistent with Ethiopian findings (35).

Furthermore, households with Frequency of water supply with more than once a day and once in two weeks had significantly 2.70 litres and 5.127 litres lower changes on level of per capita water consumption than the 24 h continuous supply, respectively. Thi is due to People usually tended to use a lot of water as long as it is available. At medium and bad water supply continuity, the discontinuity of water forced people to use lesser amount. This finding is in line with the study done in Iraq (8).

## 5 Conclusion and recommendation

### 5.1 Conclusion

The study assessed domestic water consumption at the household level in rural Harari regional state, Eastern Ethiopia. A total of 385 households were randomly selected, with the majority relying on public taps (41.3%) and hand-dug wells (16.2%). The average daily water consumption was 103.3 liters per house holdper day and 17.6 liters per person per day, which is less than 20 L/c/d of WHO basic standards. Furthermore, multiple linear regressions revealed six determinants of household water consumption: household size, time of collection, price of water, frequency of water supply, main source of water, and wealth status. The analysis explains R^2^ of 0.592, implying 59.1% of the variation in domestic per capita water consumption in the rural Harari region, highlighting the need for policy-making to ensure basic water requirements for rural households.

### 5.2 Recommendation

The study suggests that a coordinated effort between government and non-governmental organizations is needed to achieve the minimum recommended per capita water consumption. It recommends constructing more protected water sources near households and increasing the reliability of existing main sources to reduce unimproved sources and waiting times. Local administrators should also consider socio-demographic and economic factors in demand management strategies. The findings are limited to water sourced from sources and consumed at home, and future studies should consider accurate measurements of water consumption by rural households.

## Data Availability

The original contributions presented in the study are included in the article/Supplementary material, further inquiries can be directed to the corresponding author/s.
