# Supplementary material for: Domestic water consumptions and associated factors in rural household of Harari region, Eastern Ethiopia; A cross sectional study
Source: Front Public Health. 2025 Jan 16;12:1395946. doi: 10.3389/fpubh.2024.1395946 (PMC11781252; doi:10.3389/fpubh.2024.1395946)
Supplement: Supplementary file 1 [file Data_Sheet_1.docx]

## 2.3 Conceptual Frameworks

Figure 1: conceptual frame work for assessment of factors affecting domestic water consumption (developed from literature review).

Randomly

166 sample

139 sample

Systematic

80 sample

385 sample

Figure 2.Sampling procedure

**Proportion of woreda samples**

**Sofi woreda** = sofi HH *385/ Total HH = 12,576*385/29,232= 166 sample

Total selected kebeles HH = 1408 +1282+1023= 3713 HH

from Gelmashira = 1408*166/3713= 63 HH

from Burke =1282*166/3713 = 57 HH

from Kile = 1023*166/3713 = 46 HH

**Dire teyere**= 10,565*385/29,232 = 139 sample

Three selected kebeles THH = 1619+1042+1408= 4069 HH

Abubeker mute =1619*139/4069 = 55 HH

Hassengey =1042*139/4069 = 36 HH

Sigecha = 1408*139/4069 = 48 HH

**Erer woreda** = 6091*385/29,232= 80 sample

Two selected kebel = 913 +2498 = 3411 HH

Dodota = 913*80/3411= 21 HH

Weldeya =2498*80/3411 = 59 HH

Table 7: total average and average per capita water consumption of househod in rural Harari region, Eastern Ethiopia, November 2022.

|  | | | | | |
| --- | --- | --- | --- | --- | --- |
|  | N | Minimum | Maximum | Mean | Std. Deviation |
| HHWC | 383 | 20 | 220 | 103.3 | 40.38 |
| Per capita | 383 | 5.71 | 40.00 | 17.6 | 6.9 |
| N | 383 |  |  |  |  |

Table 8: Model summary

| **Model Summary^b^** | | | | | |
| --- | --- | --- | --- | --- | --- |
| Model | R | R Square | Adjusted R Square | Std. Error of the Estimate | Durbin-Watson |
| 1 | .769^a^ | .591 | .498 | 4.56760 | 2.362 |

Table 9: ANOVA Table

| **ANOVA^a^** | | | | | | |
| --- | --- | --- | --- | --- | --- | --- |
| Model | | Sum of Squares | df | Mean Square | F | Sig. |
| 1 | Regression | 3166.278 | 24 | 131.928 | 6.324 | .000^b^ |
|  | Residual | 2190.616 | 105 | 20.863 |  |  |
|  | Total | 5356.894 | 129 |  |  |  |
| a. Dependent Variable: per capita water consumption | | | | | | |
